# Geochemical Characteristics and Significance of Organic Matter in Hydrate-Bearing Sediments from Shenhu Area, South China Sea

**DOI:** 10.3390/molecules27082533

**Published:** 2022-04-14

**Authors:** Yuanyuan Li, Lei Pang, Zuodong Wang, Qianxiang Meng, Ping Guan, Xuemin Xu, Yunxin Fang, Hailong Lu, Jianliang Ye, Wenwei Xie

**Affiliations:** 1 College of Engineering, Peking University, Beijing 100871, China; 1801111693@pku.edu.cn; 2 Beijing International Center for Gas Hydrate, School of Earth and Space Sciences, Peking University, Beijing 100871, China; lei.pang@epfl.ch (L.P.); pguanl@pku.edu.cn (P.G.); 3 Southern Marine Science and Engineering Guangdong Laboratory, Guangzhou 511458, China; 4 Key Laboratory of Petroleum Resource Research, Northwest Institute of Eco-Environment & Resources, Chinese Academy of Sciences, Lanzhou 730000, China; wzd@lzb.ac.cn (Z.W.); mengqx@lzb.ac.cn (Q.M.); 5 National Research Center for Geoanalysis, Beijing 100037, China; xueminxu_cup@126.com; 6 MLR Key Laboratory of Marine Mineral Resources, Guangzhou Marine Geological Survey, Ministry of Land and Resources, Guangzhou 510075, China; jianliangye@hydz.cn (J.Y.); 13700368287@139.com (W.X.)

**Keywords:** organic matter, biomarkers, Rock-Eval, gas hydrate, biodegradation, South China Sea

## Abstract

Rock-Eval pyrolysis and the biomarker composition of organic matter were systematically studied in hydrate-bearing sediments from the Shenhu area, South China Sea. The *n*-alkane distribution patterns revealed that the organic matter in the sediments appeared to originate from mixed sources of marine autochthonous input, terrestrial higher plants, and ancient reworked organic matter. The low total organic carbon contents (average < 0.5%) and the low hydrogen index (HI, <80 mg HC/g TOC) suggested the poor hydrocarbon-generation potential of the deposited organic matter at a surrounding temperature of <20 °C in unconsolidated sediments. The abnormally high production index and the fossil-originated unresolved complex mixture (UCM) accompanied by sterane and hopane of high maturity indicated the contribution of deep hydrocarbon reservoirs. Preliminary oil-to-source correlation for the extracts implied that the allochthonous hydrocarbons in the W01B and W02B sediments might have originated from the terrestrial source rocks of mature Enping and Wenchang formations, while those of W03B seem to be derived from more reduced and immature marine source rocks such as the Zhuhai formation. The results of the organic extracts supported the previous identification of source rocks based on the isotopic composition of C_2+_ hydrate-bound gases. The biomarker of methanogens, squalane, was recognized in the sediments of this study, possibly suggesting the generation of secondary microbial gases which are coupled with the biodegradation of the deep allochthonous hydrocarbons.

## 1. Introduction

Natural gas hydrates are crystalline compounds composed of water and gases formed under high pressure and low temperature [1], which have been a frontier issue in both the industrial and academic research fields [2,3]. Marine geological surveys and drilling expeditions have shown that the Shenhu area, South China Sea (SCS) has abundant gas hydrate resources [4,5,6,7], and it has been one of the most promising exploration areas for gas hydrates [8,9,10,11,12,13].

It is conducive to understanding the formation mechanism, evolution history, and distribution of gas hydrate to acknowledge the origins and sources of hydrate-bound gas and its corresponding source rocks [3,14,15]. At present, most studies rely on the geochemical characteristics of gas hydrate, which provide limited available indicators (mainly carbon and hydrogen isotopes of methane (δ^13^C-CH_4_ and δD-CH_4_) and the dry coefficient (C_1_/(C_2_ + C_3_); C_1_, C_2_, and C_3_ refer to methane, ethane, and propane, respectively) to study the gas origins [16,17,18]—the ambiguous boundaries in the genetic diagrams and the complex secondary processes present difficulties for a definite interpretation. Fortunately, biomarkers extracted from the hydrate-bearing sediment have more proxies and thereby provide more information regarding the original deposition and potential oil-source correlation of organic matter, and more clues as to the source rocks of the hydrate-bound gas.

**Figure 1:**
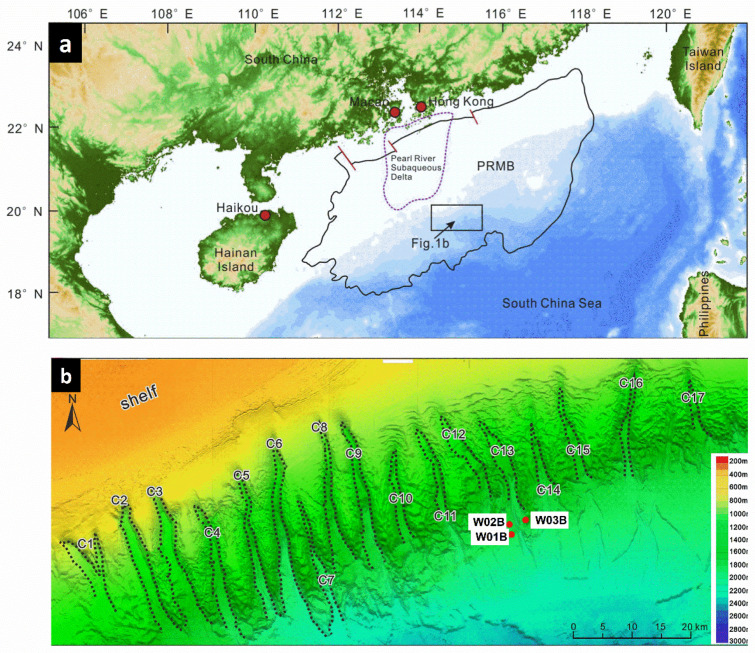
(**a**) Location of the Shenhu area in the Baiyun Sag of the Pearl River Mouth Basin (PRMB), South China Sea (SCS), modified from [24]; (**b**) location of the hydrate coring sites of this study—W01B, W02B, and W03B. C1–C17 refer to 17 canyons in the Shenhu area, modified from [10,21,25].

The geochemical characteristics of gases bound in hydrates and core sediments at the three sites used in this study have been previously reported [19,20,21,22]. Hydrate samples from W03B were suggested to be dominated by microbial gases, while those from W01B and W02B exhibited a high content of thermogenic hydrocarbons [21,22]. Therefore, it is an intriguing phenomenon that these three drilling sites have different gas origins and sources, despite the fact that they are adjacent and located on either side of a canyon in the Shenhu area (Figure 1b). Moreover, for the Shenhu area, it has been reported that the deep conventional hydrocarbons have undergone an upward migration and subsequent microbial biodegradation to participate in the formation of shallower gas hydrate, based on the gas geochemistry and microbial community [21,22,23], but this inference still lacks further robust evidence. Remarkably, biomarker patterns are important evidence to shed new light on the contribution of petroleum hydrocarbons from deep oil/gas reservoirs and provide clues for potential oil/source correlation. Apart from this, lipid biomarkers in gas hydrate systems may provide important information for microbial communities associated with methane metabolic activities.

This study investigates the organic biomarker and pyrolysis geochemistry of sediment samples from three sites (W01B, W02B, and W03B) in the Shenhu area by Gas Chromatography–Mass Spectrometry (GC–MS) and Rock-Eval analyses to determine: (1) the sources of the organic matter in the sediments; (2) the microbial gas generation potential of the in situ sediment; (3) the existence/absence of a deep hydrocarbon contribution and potential oil-source correlation; and (4) the significances of organic matter for the gas hydrate system research in the Shenhu area. The novel results of this study might provide profound insights into gas hydrate formation in the Shenhu area.

## 2. Geological Setting

**Figure 2:**
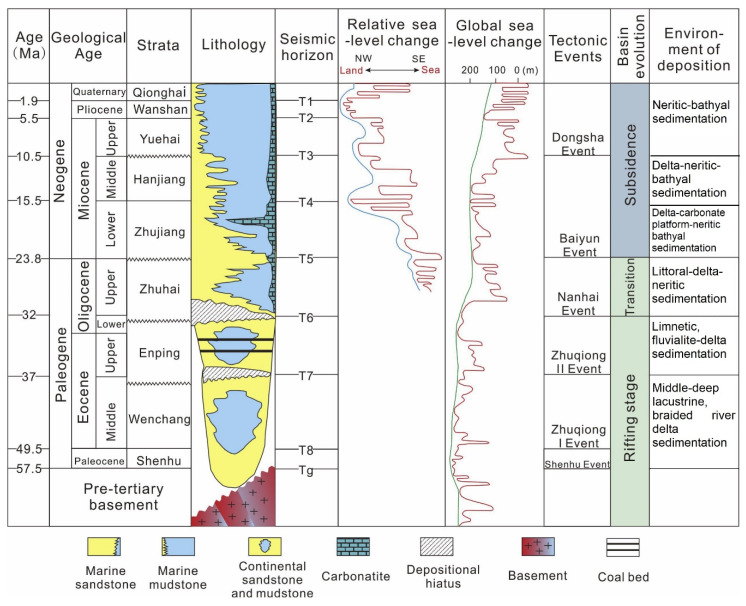
Structural evolution characteristics and stratigraphic column of the PRMB, modified from [31,47].

Located at the intersection of three tectonic plates, the SCS is the largest passive marginal sea in the Western Pacific [26,27]. The complex history of tectonic evolution and the promising prospects of oil and gas have made the SCS a natural laboratory for marine geology research [28,29]. The Shenhu drilling area is located in the Baiyun Sag, a deep-water depression in the Pearl River Mouth Basin (PRMB) of the SCS [30,31] (Figure 1), which has favorable conditions for gas hydrate formation and preservation [32,33,34,35]. High-quality source rocks have been found to supply gas for the shallow gas hydrate deposits [11,12,36,37,38]. The Eocene Wenchang formation and the Oligocene Enping formation are considered to be mature/overmature source rocks for the generation of thermogenic gases (Figure 2) [11,37,38]. The Zhujiang formation and the Hanjiang formation cannot provide thermogenic gas due to the limitation of their maturity, but they can still provide microbial gas as source rocks (Figure 2) [12,36]. Conventional oil/gas reservoirs have been explored in the PRMB, further confirming the great resource potential of this area [11,39,40,41]. Furthermore, polygonal faults induced by the overpressure environment of rapid deposition and large-scale mud diapirs formed during the neotectonic movement, all providing migration channels for gases accumulating at the gas hydrate stability zone (GHSZ) [32]. In addition, regional exploration shows that the Baiyun Sag has favorable geological conditions (seafloor pressures greater than 10 MPa, seafloor temperatures below 4 °C, and a geothermal gradient of 45–67.7 °C/km) for gas hydrate preservation [42,43]. Furthermore, it is noteworthy that the Shenhu area is the first place in the world where high concentrations of disseminated natural gas hydrate have been discovered in fine-grain sediment [9,25,44,45,46]. The gas hydrate is finely distributed in the foram-rich clayed silts, with a concentration of 20–60% of the pore volume [9,25,44].

## 3. Results

### 3.1. Rock-Eval Pyrolysis

**Figure 3:**
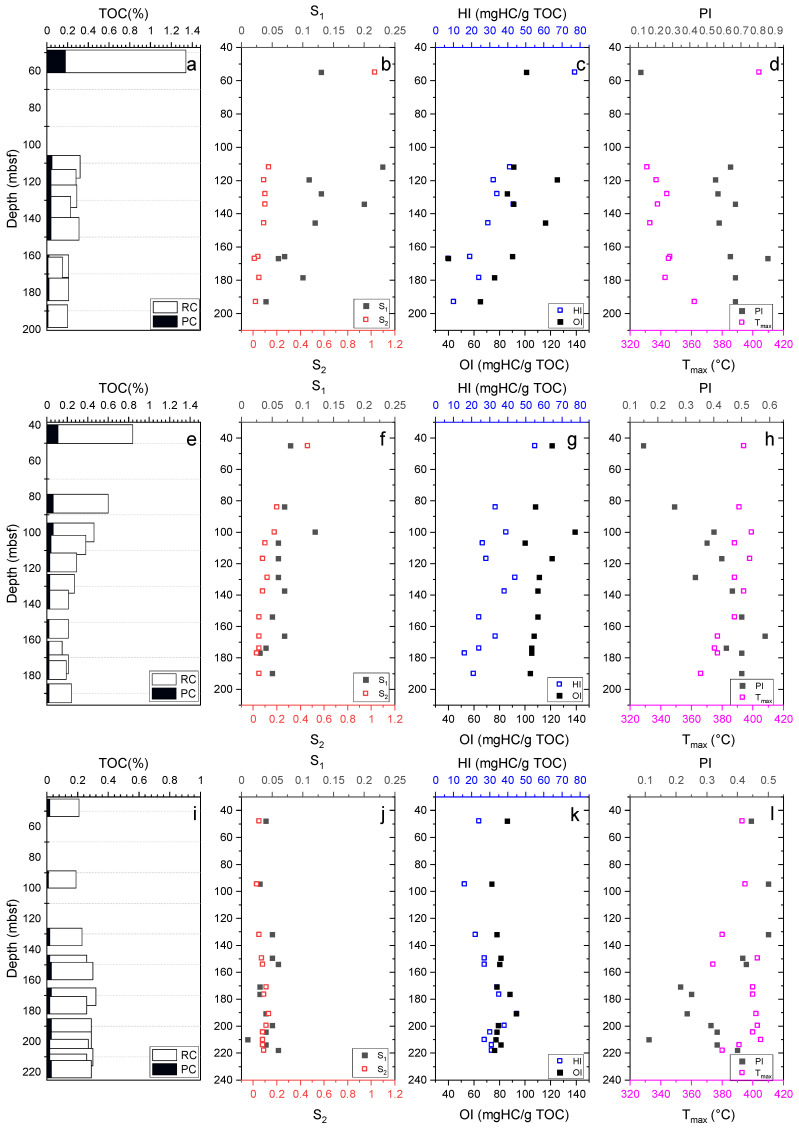
Depth profile of total organic carbon (TOC) (including pyrolysable organic carbon (PC) and residual organic carbon (RC)), free hydrocarbons (S_1_), oil potential (S_2_), hydrogen index (HI), oxygen index (OI), production index (PI), and T_max_ of W01B (**a**–**d**), W02B (**e**–**h**), and W03B (**i**–**l**).

The total organic carbon (TOC) contents of sediments from W01B and W02B decreased exponentially with depth from 1.34% to 0.15% (Figure 3a,e), while that of W03B slightly increased with depth from 0.19% to 0.32% (Figure 3i). The S_1_ and S_2_ of sediments from the three sites all showed extremely low values, and the depth variation profiles were similar to those of the TOC (Figure 3b,f,j). The HI values of sediments from the three sites ranged from 7 to 77, 16 to 55, and 16 to 45 mg HC/g TOC, respectively (Figure 3c,g,k). The OI indexes of sediments from the three sites were in the range of 40–125, 100–139, and 74–93 mg HC/g TOC, respectively (Figure 3c,g,k). The production indexes (PIs) of sediments from the three sites were in the range of 0.11–0.86, 0.15–0.58, and 0.11–0.5, respectively (Figure 3d,h,l). The T_max_ values of the sediments were in the range of 331–404 °C, 366–399 °C, and 374–405 °C, respectively (Figure 3d,h,l). A detailed data composition is shown in Table S1.

### 3.2. Biomarkers

#### 3.2.1. *n*-Alkanes

**Figure 4:**
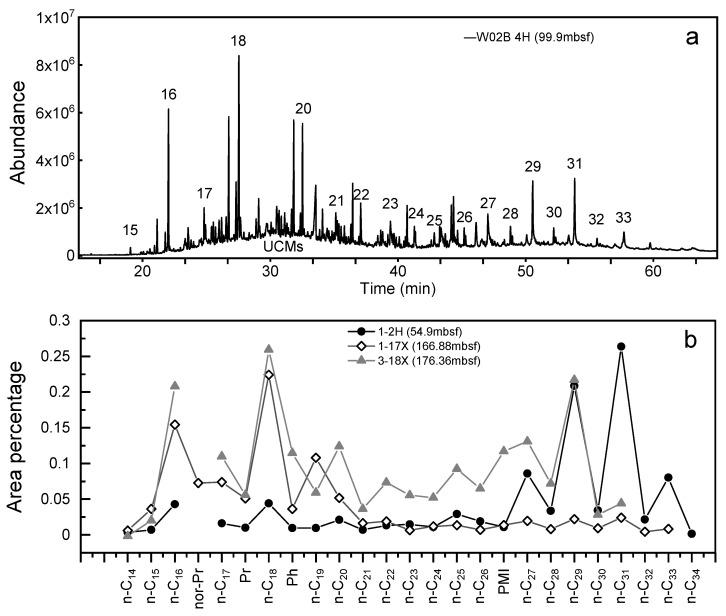
(**a**) Total ion chromatogram (TIC) of extracts from the studied sample W02B-4H (99.9 mbsf); (**b**) representative *n*-alkane composition from W01B-2H (54.9 mbsf), W01B-17X (166.88 mbsf), and W03B-18X (176.36 mbsf).

The samples obtained from the three sites mainly contained *n*-alkanes from *n*-C_12_ to *n*-C_35_, and most of the samples displayed a bimodal distribution pattern (Figure 4) with a maximum at *n*-C_16_ or *n*-C_18_ of the pre-peak and *n*-C_29_ or *n*-C_31_ of the post-peak (Figure 4a). Most samples showed a bimodal distribution pattern with the pre-peak dominating (e.g., Figure 4b: 3-18X, 1-17X), except for the shallow samples, which showed the post-peak dominating (e.g., Figure 4b: 1-2H). The pre-peak showed an even-carbon dominance, while the post-peak showed an odd-carbon dominance (Figure 4a).

#### 3.2.2. Isoprenoid

The two major acyclic isoprenoid hydrocarbons in all the samples were pristane and phytane. Empirical observations of marginally mature to mature rocks suggest that the ratios of pristane to phytane vary systematically according to the type of depositional environment [48,49]. The sources of pristane and phytane are multiple and complex, but, as a general rule, Pr/Ph values > 3 are typical of organic matter with predominantly terrestrial sources deposited in or transported through an anoxic environment; values of less than 1 are typically found in sediments from anoxic marine or hypersaline depositional environments [48,50,51]. The Pr/Ph of samples from the three sites ranged from 0.6 to 1.11, 0.61 to 0.97, and 0.18 to 0.8, respectively; all were less than 1 except for W01B-10x (1.11) and W01B-17x (1.04), indicating a phytane-dominant reduction depositional environment. Except for Pr and Ph, another kind of isoprenoid, squalene, was detected in samples from all three sites, of which the content ranged from 1.38 to 10.06, 2.49 to 7.84, and 3.71 to 13.19, respectively. The total squalane content of W03B was higher than that of W01B and W02B.

#### 3.2.3. Steranes and Terpanes

**Figure 5:**
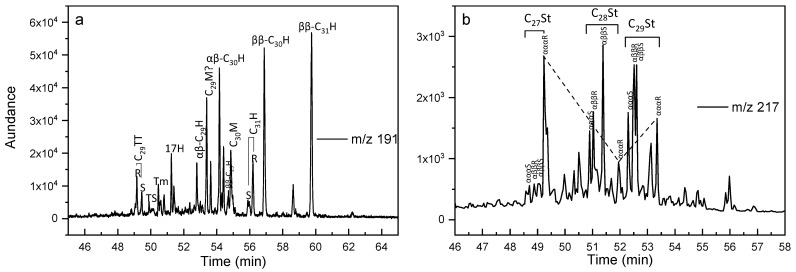
(**a**) Distributions of terpane and hopanes on the mass chromatograms (m/z 191). Peak assignments: TT—tricyclic terpane; H—hopane; M—moretane; Ts—18α(H)22,29,30-trisnorhopane; and Tm—17α(H)-22,29,30-trisnorhopane. R, S: the configuration of the compounds. (**b**) Distributions of the diasteranes and regular steranes on the mass chromatograms (m/z 217).

A series of terpanes were detected in the extracts, including tricyclic terpanes (TT), tetracyclic terpanes (TeT), and hopanes series (Figure 5a). Figure 5a shows the distribution patterns of the hopane series. They are characterized by the predominance of C_30_ hopane (C_30_H) and C_29_H. The relatively high abundance of biological configurational isomers 17β(H), 21β(H)-hopane (ββ-C30H) and 17β(H), 21β(H)-homohopane (ββ-C_31_H) indicate their low maturation levels (Figure 5a). The 18α(H)-22,29,30trisnorhopane (Ts) had a relatively high abundance in all samples, with Ts/Tm ratios ranging from 0.25 to 0.81, 0.19 to 0.72, and 0.11 to 0.55, respectively. C_31_22S/(S + R) showed less variation than Ts/Tm, with values of 0.23~0.53, 0.21~0.4, and 0.1~0.21, respectively, whereas gammacerane (Ga), oleanane (OL), and C_33_–C_35_ homohopanes were absent. C_27_ diasteranes and C_27_, C_28_, and C_29_ regular steranes were detected in the extracts (Figure 5b). The C_27_, C_28_, and C_29_-5α(H),14α(H),17α(H)-20R-cholestanes exhibited an approximately V-shaped distribution pattern (Figure 5b). The sterane maturation parameter αααC_29_20S/20(S + R) index of the three sites ranged from 0.16 to 0.43, 0.15 to 0.36, and 0.03 to 0.24, respectively, and the C_29_ββ/ββ + αα index of the three sites ranged from 0.24 to 0.42, 0.23 to 0.43, and 0.07 to 0.49, respectively.

## 4. Discussion

### 4.1. Sources and Preservation of Organic Matter

#### 4.1.1. *n*-Alkanes

It is thought that the light hydrocarbons (C_21−_) are mainly derived from the lipids of marine algae, while the long-chain hydrocarbons (C_21+_) mainly originate from the waxes of terrestrial higher vascular plants and the lipid fragments derived from conifers [52,53,54]; thus, the bimodal distribution pattern of most of the samples in this study indicates a terrestrial–marine mixed source (Figure 4a,b), except for the layers such as W01B-10X and W01-17X, which tended to be unimodal pre-peak (Figure 4b), and layers such as W01B-2H and W02B-1H, which had an obvious unimodal post-peak (Figure 4b). The TAR, C_21-_/C_21+_, and OEP_2_ showed that W01B and W02B had more terrestrial contributions in the shallow part (Table S1), while W03B had the opposite tendency, which may be related to the geographical location of the three sites. W01B, W02B, and W03B were located on either side of the migration canyon in the Shenhu area, respectively, and formed a unique sedimentary pattern under the complex interaction of a gravity current, a bottom current, and internal waves, resulting in differences in deposition between the sites [55,56,57,58,59,60].

In addition to the mixed-source characteristics, it was remarkable that C_12_-C_22_ *n*-alkanes with even-to-odd predominance were observed in all samples (Figure 4). They were also observed in recent/ancient sediments as well as in the marine sediments [61,62,63,64,65]. The possible reasons for the even–odd dominance of short-chain *n*-alkanes (C_12_-C_22_) in the sediments are as follows: (I) normal fatty acids with odd-carbon predominance were reduced in a strongly reducing environment during deposition [66]; (II) anthropogenic pollution from fossil fuels or hydrocarbon leakage from the underlying high-maturity formations [67]; (III) biomass combustion can also form the dominant distribution of normal short-chain carbon pairs (thermal degradation of long-chain *n*-alkanes to short-chain *n*-alkanes) [68]; and (IV) they are derived from specific species of bacteria or fungi [62,69,70]. All the layers of the three sites displayed obvious even-carbon predominance, which is presumed to be independent of hydrocarbon leakage but usually indicates the direct input of microorganisms in sediments.

Compared to short-chain *n*-alkanes, long-chain C_23_-C_33_ *n*-alkanes with a strong odd–even carbon preference (particularly C_27_, C_29_, and C_31_) are indicative of terrestrially derived organic matter [71]. On the other hand, *n*-alkane distributions without odd/even predominance in sediments lacking petrogenic markers (hopane and sterane mixtures specific to mature organic matter) have been attributed to microbial odd-C-numbered inputs or to the microbial reworking of the *n*-alkane mixtures [70,72,73,74], suggesting that they can be used as tracers for degraded or microbially altered organic matter [75,76]. Both types of *n*-alkane distribution were quantified separately in the sediment from the three sites using the following equations [77,78]:

(1) $$A_{\mathrm{odd}}=\overset{6}{\underset{i=1}{\sum }}C_{\left(21+2i\right)}-\overset{5}{\underset{i=1}{\sum }}C_{\left(22+2i\right)}-\frac{C_{22}+C_{32}}{2}$$

(2) $$A_{\mathrm{rew}}=C_{t}-A_{\mathrm{odd}}$$

where C_i_ is the measured concentration of each *n*-alkane and C_t_ is the sum of all *n*-alkane homologues between C_21_ and C_33_. A_rew_ refers to n-alkanes without an even/odd carbon number preference and A_odd_ refers to odd carbon number dominant n-alkanes.

**Figure 6:**
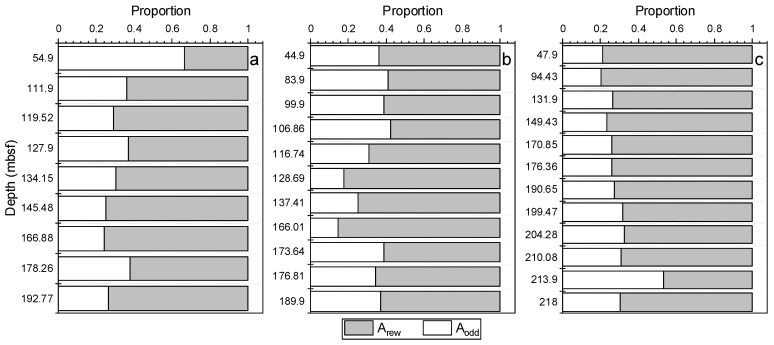
Relative proportion of n-alkanes without an even/odd carbon number preference (A_rew_) and odd carbon number dominant n-alkanes (A_odd_) of *n*-alkanes in sediment samples from (**a**) W01B, (**b**) W02B, and (**c**) W03B. Blank area and shaded area represent A_odd_ and A_rew_, respectively.

From the calculation results, W01B and W02B were shown to have more contributions from terrestrial plants in the shallow part and more contributions from reworked organic matter in the deep part (Figure 6a,b). However, the proportion of W03B was relatively constant with depth and had the highest contribution from reworked organic matter among the three sites (Figure 6c). From the perspective of *n*-alkanes, organic matter from the Shenhu area appeared to be from mixed marine, continental, and ancient reworked organic matter sources, with the latter possibly contributed by turbidity flows/bottom currents from the upper continental shelf or nearby continental slope.

#### 4.1.2. Steranes

Information regarding source type may sometimes be obtained from the relative abundance of C_27_:C_28_:C_29_ sterols or their fossil counterparts, the steranes [79,80]. Land plant influxes usually result in a dominance of C_29_ desmethylsteranes, whereas in predominantly marine sediments, different types and proportions of algal to zooplankton sources can result in a wide variety of sterane distributions [50,81,82]. 

C_27_/C_29_ indicates that the marine contribution of organic matter in W03B was higher than that of W01B and W02B. However, in some horizons, such as W01B, there was an increase in the pre-peak of the *n*-alkane distribution (low carbon number), but there was no corresponding reflection in the C_27_/C_29_ indicators. In this case, we suggest that the *n*-alkane distributions better reflect the terrestrial influxes.

#### 4.1.3. Pyrolysis Indexes

The Rock–Eval method developed by Espitalie et al. [83] is a widely used method for petroleum source-rock characterization and evaluation [84,85,86]. The HI, which correlates strongly with the elemental H/C ratio of the kerogen, is used to determine the hydrogen richness of a rock or sediment in order to identify the type of organic matter originally deposited in a given setting and to infer some aspects of the preservation of the remaining organic matter. High HI values are interpreted to reflect both algal and planktonic (high H/C) sources and the good preservation of the deposited organic matter [83], whereas low HI values are used to identify terrestrial organic matter sources and/or the extensive post-depositional alteration (oxidation) of algal remains [87]. Specifically, in immature marine sediments containing mixed phytoplankton, zooplankton, and bacterial debris (Type II organic matter), typical HI values range between 200 and 400 mg HC/g TOC [83], whereas in sediments containing terrestrial organic matter (Type III), the HI values are less than 200 mg HC/g TOC. Very low HI values are observed in sediments containing oxidized and reworked organic matter (Type IV) [85]. Consequently, the sediment records of HI provide information on both the sources and post-deposit processes of organic matter.

**Figure 7:**
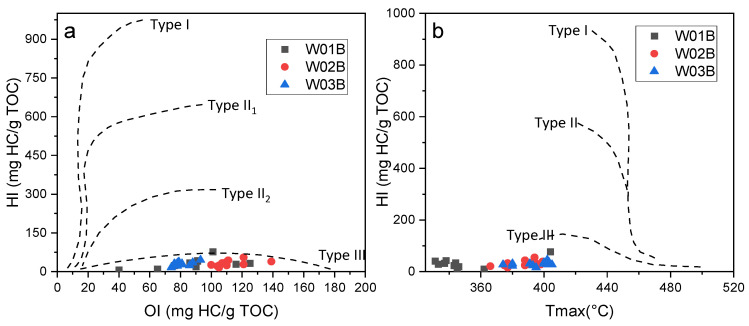
(**a**) Diagram of Rock-Eval HI vs. OI; (**b**) plot of HI vs. T_max_ of sediment samples from W01B, W02B, and W03B.

Cross plots of the HI, OI, and T_max_ of the sediment samples from the three sites are shown in Figure 6 [85]. These plots may be used similarly to a van Krevelen diagram to differentiate organic matter types. The samples in this study fell into the category of Type IV organic matter due to a lower HI (Figure 7a, b). A low HI index has been attributed to: I. A mineral matrix effect, if the sediment has variable amounts of carbonate and clay minerals, which will result in low but variable HI values [85,88,89]. However, the carbonate fraction was removed before the Rock-Eval analysis, and the clay mineral content was confirmed to be low and constant among the different samples in this study (unpublished data), so the mineral matrix effect was not considered to be the dominant factor for the low HI. II. Additional contribution from varying amounts of hydrogen-poor, reworked organic matter. Sediment reworking can increase the relative proportion of terrestrial organic matter by preferentially removing algal remains and lower the HI values by increasing the degradation degree of organic matter during post-depositional winnowing. The relatively lower HI/OI (<0.5) can also help to reconfirm this circumstance; a low HI and HI/OI in this study indicated a contribution from highly degraded reworked organic matter, since fresh or not highly degraded organic matter usually has a HI/OI greater than 2. 

The low HI value is consistent with the previously calculated ancient reworked organic matter proportion in the samples. Specifically, the W03B site showed a higher proportion of reworked organic matter and also had the highest T_max_ and lowest HI values of the three sites (Figure 7b). The explanation for this phenomenon relies on the high proportion of reworked allochthonous organic matter, which manifested itself as a high T_max_ value and a low HI value, while the immature autochthonous component was responsible for the opposite signature. 

### 4.2. The Potential of Microbial Gas Generation in Hydrate-Bearing Sediments 

In the diagenesis stage (also called the immature stage), the microbial gas generation potential of sediments is mainly controlled by the TOC content [90,91,92,93]. The formation of gas hydrate with low saturation (1–2%) from in situ sediments requires a minimum TOC content of 0.5% [93,94,95]. For the formation of high-saturation hydrate (>3%), it is not sufficient to rely solely on the biodegradation of particulate organic matter [96]. In addition, an optimal temperature (35–65 °C) is required during microbial methanogenesis activities for methanogens to survive and produce methane efficiently [90,93]. In a word, the high yield of microbial gas requires a high abundance of reactive/labile organic matter and a favorable temperature. 

The TOC contents of the studied samples from the three sites were extremely depleted (average of 0.37% for W01B and W02B, 0.13% for W03B) and did not reach the threshold of in situ methane generation and gas hydrate formation. Additionally, as mentioned before, very low HI values were observed in the sediments from this study, which indicated the presence of oxidized and reworked organic matter (Type IV) and corresponded to a poor hydrocarbon generation potential [85]. Moreover, according to the seafloor temperature (~4 °C) and geothermal gradient (54.6–62.6 °C/km) reported by Zhang et al. [21], the temperature of the studied layers (<240 m) was calculated to be less than 20 °C, which is below the optimum temperature zone for microbial activities. Furthermore, the pyrolysis parameters S_1_, S_2_, T_max_, and HI of the sediment samples from the three sites had extremely low values (Figure 7), which were far lower than the threshold criteria for source rocks (Peters and Cassa, 1994). Therefore, such depleted TOC contents (most < 0.4%), in situ temperatures (<20 °C), and HI indexes (mostly less than 50 mg HC/g TOC) may jointly suggest a fairly poor hydrocarbon generation potential for the in situ sediments at these three sites that is insufficient to form highly saturated gas hydrate relying solely on the in situ sediment.

### 4.3. Implication of Upward Migration of Deep Hydrocarbons

#### 4.3.1. Evidence of Petroleum Hydrocarbons 

**Figure 8:**
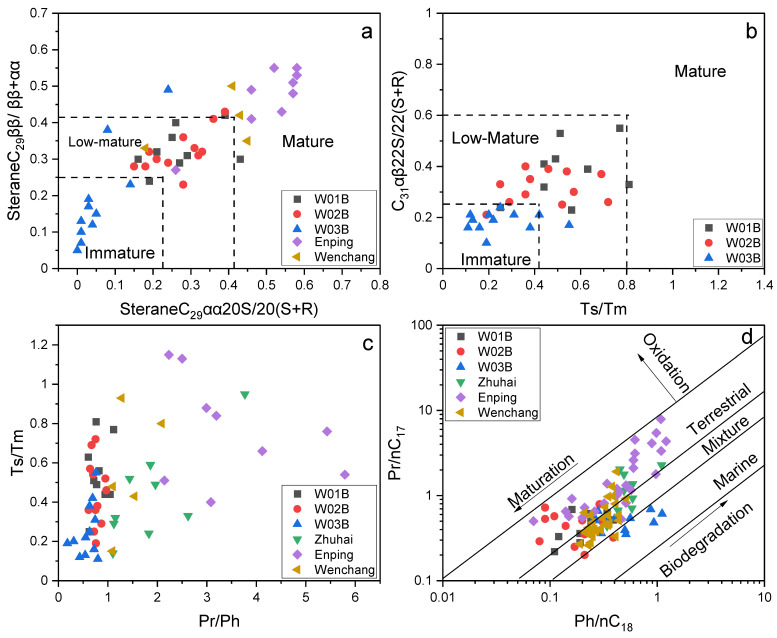
Maturity parameters of sterane (**a**) and hopane (**b**) in the sediment of W01B, W02B, and W03B. Plot of C_29_ ββ/(ββ + αα) vs. C_29_ 20S/(20S + 20R) showing the thermal maturation level of the extracts in this study and representative source rocks from the deep-water area of the Shenhu area, Baiyun Sag. Cutoff-points adopted from [51,81,102,103,104,105,106,107,108,109,110,111,112,113,114]. (**c**) Plot of Pr/Ph vs. Ts/Tm. (**d**) Plot of Ph/nC_18_ vs. Pr/nC_17_ (modified from [115]), showing the geochemical differences between the extracts in this study and representative source rocks from the Shenhu area. Geochemical parameters of representative source rock samples are cited from [116,117,118,119].

Primarily, the pyrolysis parameters (S_1_, S_2_, T_max_, and HI indexes) of the samples were significantly lower than the threshold for source rocks [97], indicating that thermogenic hydrocarbons could not be generated. However, most of the samples had a PI greater than 0.1, even as high as 0.8 (W01B-16, -17X; W02B-7H) (Figure 8d), indicating the intrusion and preservation of allochthonous hydrocarbons into the sediment [85]. 

In addition, an unresolved complex mixture (UCM) composed of a large number of isomers and homologues with complex branched chains and acyclic compounds that cannot be resolved by capillary columns was present in most samples, as shown in the chromatogram in Figure 4a [98,99]. The presence of a UCM in marine sediments is an indicator of chronic/degraded petroleum contamination [100]. Although a bacteria-derived UCM cannot be ruled out [101], the GC/MS identification of 17α (H), 21β (H)-hopanes and steranes confirmed the fossil origin of the UCM (Figure 8a,b). Conventional oil and gas fields are ubiquitous in the northern SCS, and the hydrate gas has been considered to be cogenetic with the deep conventional gas reservoir; therefore the detection of the petroleum-based contaminants in this area was not unusual. 

Moreover, the increase in the steranes and terpanes maturity index at the W01B and W02B sites also suggested the supply of deep mature hydrocarbons (Figure 8a,b [50,87,101,102,103,104,105,106,107,108,109,110,111,112,113]). The thermal effects of the stereochemical complexity of the basic skeleton of steranes and terpanes, i.e., the C_29_20S/(20S + 20R), C_29_ββ/(ββ + αα), Ts/Tm, and C_31_αβ-22S/22(S + R), could help to determine the maturity and the cut-off points between the immature, low-mature, and mature stages, which are labeled in Figure 8a,b [51,81,102,103,104,105,106,107,108,109,110,111,112,113,114]. The sterane and terpane maturity parameters of the samples from sites W01B and W02B of this study indicated that the organic matter mainly originated from low-mature to mature source rocks.

#### 4.3.2. Preliminary Oil-to-Source Correlation in the Extracts

Previous studies have suggested that the Baiyun Sag contains three sets of potential source rocks: Wenchang formation lacustrine source rocks, Enping formation transitional source rocks, and Zhuhai formation marine source rocks [11,21,41,116,120,121]. The first two sets of source rocks are identified as the principal source rocks, as they have a relatively high hydrocarbon generation potential and are mainly in the high-mature stage. The Zhuhai formation source rock is not considered as a primary source rock, because of its low thermal maturity [11,41,122,123]. The source rocks of the Wenchang formation Group formed in relatively anoxic to suboxic, lacustrine conditions, while the source rocks of the Enping formation were deposited in oxic, shallow lacustrine settings, with a relatively high input of terrigenous higher plants at the beginning and subsequent impact from a marine transgression; the source rocks of the Zhuhai formation were deposited in anoxic, relatively stable, neritic environments, with a greater contribution from aquatic algae relative to terrestrial higher plants [116,117].

To further investigate the origin of the allochthonous hydrocarbons within the extracts, a preliminary oil-to-source correlation was carried out based on the geochemical results obtained in this study and the geochemical parameters of the representative source rocks in the Baiyun sag reported in the literature. A series of biomarker compounds from the mature source rocks were also detected in the extracts, except for the immature hydrocarbons, such as the isoprenoids (Pr and Ph) on the mass chromatograms (Figure 4a), tricyclic terpanes and hopanes (Figure 5a), and diasteranes and regular steranes (Figure 5b). The distributions and characteristics of these biomarkers were useful for illustrating the origin of the allochthonous hydrocarbons within the extracts. In this study, Pr and Ph were detected in all samples, and the Pr/Ph ratios ranged from 0.18 to 1.11 (Figure 8c), suggesting that the organic matter sources of the allochthonous hydrocarbons were mainly deposited in an anoxic depositional environment [48,50,51]. The plot of Ph/nC_18_ versus Pr/nC_17_ (Figure 8d) shows that W01B and W02B had more terrestrial organic matter under more oxidized conditions, while W03B indicated more marine sources and a more reduced depositional environment. W01B and W02B exhibited geochemical characteristics similar to those of the terrestrial source rocks of the Enping and Wenchang formations, of which the latter contributed more to the supply. While W03B displayed geochemical characteristics similar to the marine source rocks of the Zhuhai formation.

Combined with the abnormally high maturity and the geochemical characteristics similar to those of deep mature source rocks, it is speculated that the allochthonous hydrocarbons within the extracts of W01B and W02B may include contributions from the Enping and Wenchang formations, of which the latter has a higher similarity, while W03B has a greater contribution from the immature marine source rocks of the Zhuhai formation.

### 4.4. Implication for Hydrate-Bound Gas Origin

#### 4.4.1. Evidence of Thermogenic Gas Contribution

The combination of a poor microbial gas generation potential and a weak biodegradation degree both indicate that the in situ organic matter could not generate sufficient gases for the formation of hydrate with a saturation as high as 20–60% at the three sites [25], indicating that there must be other gas sources supplying the hydrate system in this area; this is also consistent with our above speculation as to deep hydrocarbon leakage. 

**Figure 9:**
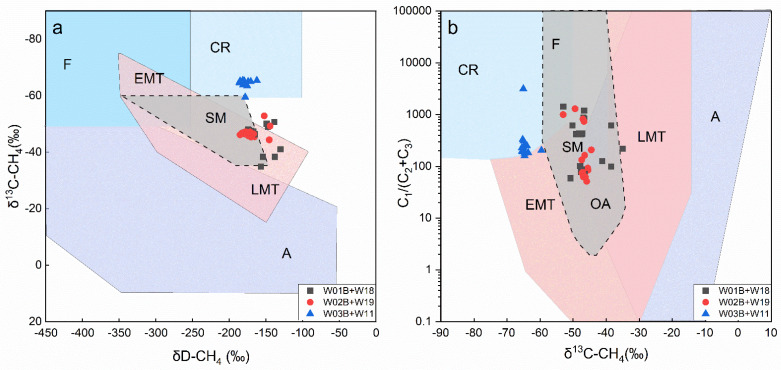
Genetic diagram of (**a**) δD-CH_4_ versus δ^13^C-CH_4_ and (**b**) C_1_/(C_2_ + C_3_) versus δ^13^C-CH_4_ (the boundary lines are from [130]), showing the genetic type of the hydrate-related gas from sites W01B, W02B, and W03B. Data from [19,20,21,22]. A—abiotic; F—fermentation; CR—CO2 reduction; EMT—early-mature thermogenic; LMT—late-mature thermogenic; SM—secondary microbial.

From the perspective of gas geochemistry, it has been suggested that the hydrate-bounded gases of sites W01B and W02B are mixed gases dominated by thermogenic gases [19,20,21,22], while the gas origin of W03B is mainly microbial gases produced by the biodegradation of organic matter in shallow strata (Figure 9a,b) [21], which further enhances the speculation that W01B and W02B received contributions from deeper thermogenic hydrocarbons. Liang et al. [22] obtained the isotope composition of the C_2+_ hydrocarbons bound in hydrate in the Shenhu area to identify the source rocks of the gas hydrate and found that the biogenic hydrocarbons and thermogenic hydrocarbons were derived from marine organic matter and terrestrial organic matter, respectively. The source rocks of thermogenic hydrate gas are interpreted to be from both the gas-prone coal strata of the Enping formation and the oil-prone medium-deep lacustrine strata of the Wenchang formation, the latter of which contributed more to the hydrocarbon supply of the gas hydrates [22]. These conclusions are consistent with the results of the oil-to-source correlation via organic biomarkers in this study. In addition, some studies show that the source rock of the Wenchang formation has been in the stage of pyrolytic dry-gas production since the early Pliocene [117,124] and is considered to be the main source rock for hydrate accumulation in this region, which is consistent with the conclusion drawn as to the extract sources in this study and the gas sources in the previous study.

#### 4.4.2. Evidence of Secondary Microbial Gas

Squalene was detected in this study, which has been used as a biomarker for methanogens [48]. A study by Lin et al. [23] verified that most abundant archaeal genera are potential methanogenic archaea in the Shenhu area, and the hydrogenotrophic type was inferred to be predominant among the multiple types of methanogenesis. It is well-known that microbial biodegradation is coupled with the methanogenic process, which means that the presence of the methanogenic process also indicates the existence of biodegradation [100,125,126]. Considering the low hydrocarbon-generation potential and intact *n*-alkane preservation of the in situ sediment, we speculate that the methanogenesis activities are likely related to the biodegradation of deep allochthonous hydrocarbons, which is coupled with the generation of secondary microbial gases [127]. This inference corresponds to the speculation of Zhang et al. [21] that the geochemical characteristics of the hydrate-bound gas from these three sites are very similar to those of confirmed biodegraded gases [21,128,129], which is also consistent with the identified putative hydrocarbon degraders in the microbial community structure study by Lin et al. [23]. Moreover, the deviations in the temperature of methanogens from the geothermal gradient of the study area suggested the migration of organic matter, emphasizing the possibility of microbially mediated secondary microbial gas formation from deep thermogenic fluid [23]. Therefore, the gases bound in hydrate from the three sites may have complex sources, including the primary in situ microbial gases, deep thermogenic gases, and secondary microbial gases generated by the biodegradation of the leakage hydrocarbons. 

## 5. Materials and Methods

The samples were retrieved by the GMGS4 expedition, which was carried out on the geotechnical drilling vessel Fugro Voyager in 2016 [25]. The water depth of the three sites is ~1285 m, 1274 m, and 1310 m, respectively, and the drilling depth was ~234 m below seafloor (mbsf), 240 mbsf, and 222 mbsf, respectively. Detailed information and lithological description of sediment samples can be found in Table S1.

### 5.1. Rock-Eval Analysis

Rock-Eval analysis was conducted by Rock-Eval IV instrument to obtain the amount of free hydrocarbons (S_1_), the amount of hydrocarbons generated through thermal cracking of nonvolatile organic matter (S_2_), the amount of CO_2_ produced during pyrolysis of kerogen (S_3_), and the temperature at which the maximum release of hydrocarbons from cracking kerogen occurred during pyrolysis (top of S_2_, T_max_). The hydrogen index (HI) was calculated from the formula HI = 1000 × S_2_/C_org_, and oxygen index (OI) was calculated using the formula OI = 1000 × S_3_/C_org_. The pyrolysis temperature of organic matter ranged from 300 °C to 600 °C, with a rising rate of 25 °C/min.

The Rock-Eval analyses were conducted at the National Research Center for Geoanalysis, China Geological Survey (CGS). 

### 5.2. Biomarker Analysis

Around 100–200g dried and crushed samples (<100 mesh) were Soxhlet extracted for 72 h by the solvent mixture of dichloromethane (DCM) and methanol (93:7, *v*:*v*). After removing the solvents and asphaltenes by *n*-hexane, the resultant soluble fraction was separated into aliphatic hydrocarbons, aromatic hydrocarbons, and polar compounds by using the column chromatography method (alumina/silica gel column). Aliphatic hydrocarbons, aromatic hydrocarbons, and nonhydrocarbons were extracted by washing with *n*-hexane, dichloromethane, and methanol, respectively.

Aliphatic hydrocarbons were analyzed using a Gas Chromatography–Quadrupole Mass Spectrometer (GC–MS) 6890N/5973N (Agilent Technologies, Palo Alto, CA, USA), which was fitted with a DB-5 MS fused silica capillary column (J&W Scientific, Agilent, USA; 30 m × 0.25 mm × 0.25 µm). The GC oven temperature was initially set at 80 °C (hold for 5 min), then programmed to 290 °C (hold for 40 min) at a rate of 4 °C/min. The compounds were identified by comparing their mass spectra with those in the NIST02 library and published data. 

The biomarker experiments were conducted in the sample pretreatment laboratory of the Key Laboratory of Petroleum Resource Research, Northwest Institute of Eco-Environment & Resources, Chinese Academy of Sciences.

## 6. Conclusions

The organic geochemical characteristics of hydrate-bearing sediment provide clues for studying the origins and formation processes of gas hydrate. In this study, the Rock-Eval pyrolysis and biomarkers (*n*-alkane, isoprenoid, sterane, and hopane) of organic matter were systematically studied in hydrate-bearing sediments from the Shenhu area (GMGS4-W01B, -W02B, and -W03B), South China Sea. According to the experimental results, the following conclusions can be drawn.

(1) The *n*-alkane distribution patterns reveal that the organic matter in sediments from the three sites appears to originate from the mixed sources of marine, terrestrial higher plant, and ancient reworked organic matter; the presence of the latter is verified by the pyrolysis parameters, especially the low hydrogen index (HI) and high T_max_. The relative proportion of terrestrial higher plants to reworked organic matter was calculated from equations, and it was found that the depth variation trend of the ratio was consistent with that of the HI and T_max_, and the formation with a high proportion of reworked organic matter manifested as a low HI and high T_max_.
(2) The low total organic carbon (TOC) contents (average < 0.5%) with a HI < 80 mg HC/g TOC suggest the poor hydrocarbon-generation potential of the deposited organic matter at a surrounding temperature of <20 °C in unconsolidated sediments, which also indicates that the highly saturated hydrate could not have been formed solely by the in situ sedimentary organic matter.
(3) The abnormally high production index (PI), fossil-originated UCM, and sterane and hopane of high maturity indicate the contribution of deep hydrocarbon reservoirs, which is consistent with the thermogenic origin contribution of hydrate-bound gases.
(4) The preliminary oil-to-source correlation for the extracts implied that the allochthonous hydrocarbons in the W01B and W02B sediments might have originated from the low-mature to mature terrestrial source rocks of the Enping and Wenchang formations, while those of W03B seem to be derived from more reduced and immature marine source rocks, such as those of the Zhuhai formation. The results of the organic extracts indicated that some organic matter might have migrated from deep hydrocarbon reservoirs, similar to the hydrate-bound gas, and supported the previous identification of source rocks based on the isotopic composition of C_2+_.
(5) That the biomarker of methanogens, squalane, was recognized in the sediments of this study might suggest the generation of secondary microbial gases which are coupled with the biodegradation of the deep allochthonous hydrocarbons, indicating the complex component of the hydrate-bound gases.

## Data Availability

The data used to support the findings of this study are available from the corresponding author upon request.

## Supplementary Materials

The following supporting information can be downloaded at: https://www.mdpi.com/article/10.3390/molecules27082533/s1, Table S1: Site information and lithological description of the samples in this study; Table S2: Biomarker composition and Rock-Eval pyrolysis parameters of sediments in this study.

## References

[1] Sloan E.D. (2003). Fundamental principles and applications of natural gas hydrates. Nature.

[2] Collett T.S., Johnson A.H., Knapp C.C., Boswell R. (2009). Natural Gas Hydrates: A Review. AAPG Mem..

[3] Kvenvolden K.A. (1995). A review of the geochemistry of methane in natural gas hydrate. Org. Geochem..

[4] Liu H.L., Yao Y.J., Deng H. (2011). Geological and geophysical conditions for potential natural gas hydrate resources in southern South China Sea waters. J. Earth Sci. China.

[5] Trung N.N. (2012). The gas hydrate potential in the South China Sea. J. Pet. Sci. Eng..

[6] Jiang H., Pang X.Q., Shi H.S., Yu Q.H., Cao Z., Yu R., Chen D., Long Z.L., Jiang F.J. (2015). Source rock characteristics and hydrocarbon expulsion potential of the Middle Eocene Wenchang formation in the Huizhou depression, Pearl River Mouth basin, south China sea. Mar. Pet. Geol..

[7] He D., Hou D., Zhang P., Harris M., Mi J., Chen T., Li J. (2016). Reservoir characteristics in the LW3-1 structure in the deepwater area of the Baiyun sag, South China Sea. Arab. J. Geosci..

[8] Zhang X.L., He J.X., Zhao Y.D., Wu H.C., Ren Z.Q. (2017). Geochemical Characteristics and Origins of the Crude Oil of Triassic Yanchang Formation in Southwestern Yishan Slope, Ordos Basin. Int. J. Anal. Chem..

[9] Wu N.Y., Zhang H.Q., Yang S.X., Zhang G.X., Liang J.Q., Lu J.A., Su X., Schultheiss P., Holland M., Zhu Y.H. (2011). Gas Hydrate System of Shenhu Area, Northern South China Sea: Geochemical Results. J. Geol. Res..

[10] Wang X.J., Collett T.S., Lee M.W., Yang S.X., Guo Y.Q., Wu S.G. (2014). Geological controls on the occurrence of gas hydrate from core, downhole log, and seismic data in the Shenhu area, South China Sea. Mar. Geol..

[11] Zhang G.C., Yang H.C., Chen Y., Ji M., Wang K., Yang D.S., Han X.Y., Sun Y.H. (2014). The Baiyun Sag: A giant rich gas-generation sag in the deepwater area of the Pearl River Mouth Basin. Nat. Gas. Ind..

[12] Su P.B., Liang J.Q., Peng J., Zhang W., Xu J.H. (2018). Petroleum systems modeling on gas hydrate of the first experimental exploitation region in the Shenhu area, northern South China sea. J. Asian Earth Sci..

[13] Zhang W., Liang J., Wei J., Lu J., Su P., Lin L., Huang W., Guo Y., Deng W., Yang X. (2020). Geological and geophysical features of and controls on occurrence and accumulation of gas hydrates in the first offshore gas-hydrate production test region in the Shenhu area, Northern South China Sea. Mar. Pet. Geol..

[14] Collett T.S. (1993). Natural Gas Hydrates of the Prudhoe Bay and Kuparuk River Area, North Slope, Alaska. AAPG Bull..

[15] Collett T.S. (2002). Energy resource potential of natural gas hydrates. AAPG Bull..

[16] Schoell M. (1983). Genetic Characterization of Natural Gases. AAPG.

[17] Chung H.M., Gormly J.R., Squires R.M. (1988). Origin of gaseous hydrocarbons in subsurface environments: Theoretical considerations of carbon isotope distribution. Chem. Geol..

[18] Whiticar M.J. (1999). Carbon and hydrogen isotope systematics of bacterial formation and oxidation of methane. Chem. Geol..

[19] Li J., Lu J., Kang D., Ning F., Lu H., Kuang Z., Wang D., Liu C., Hu G., Wang J. (2019). Lithological characteristics and hydrocarbon gas sources of gas hydrate-bearing sediments in the Shenhu area, South China Sea: Implications from the W01B and W02B sites. Mar. Geol..

[20] Wei J., Fang Y., Lu H., Lu H., Lu J., Liang J., Yang S. (2018). Distribution and characteristics of natural gas hydrates in the Shenhu Sea Area, South China Sea. Mar. Pet. Geol..

[21] Zhang W., Liang J.Q., Wei J.G., Su P.B., Lin L., Huang W. (2019). Origin of natural gases and associated gas hydrates in the Shenhu area, northern South China Sea: Results from the China gas hydrate drilling expeditions. J. Asian Earth Sci..

[22] Liang Q., Xiao X., Zhao J., Zhang W., Li Y., Wu X., Ye J., Qin X., Qiu H., Liang J. (2022). Geochemistry and sources of hydrate-bound gas in the Shenhu area, northern south China sea: Insights from drilling and gas hydrate production tests. J. Pet. Sci. Eng..

[23] Lin G., Lu J., Luo K., Fang Y., Liu J., Ji X., Ge S., Liu J., Su M. (2022). Characterization of bacterial and archaeal community structure in deep subsurface sediments in the Shenhu area, northern South China Sea. Mar. Pet. Geol..

[24] Su M., Alves T.M., Li W., Sha Z.B., Hsiung K.-H., Liang J.Q., Kuang Z.G., Wu N.Y., Zhang B., Chiang C.-S. (2019). Reassessing two contrasting Late Miocene-Holocene stratigraphic frameworks for the Pearl River Mouth Basin, northern South China Sea. Mar. Pet. Geol..

[25] Yang S.X., Lei Y., Liang J.Q., Holland M., Schultheiss P., Lu J.A., Wei J.G. Concentrated Gas Hydrate in the Shenhu Area, South China Sea: Results From Drilling Expeditions GMGS3 & GMGS4. Proceedings of the 9th International Conference on Gas Hydrates (ICGH 2017).

[26] Sun Z., Zhou D., Zhong Z.H., Xia B., Qiu X.L., Zeng Z.X., Jiang J.Q. (2006). Research on the dynamics of the South China Sea opening: Evidence from analogue modeling. Sci. China Ser. D.

[27] Xu J.Y., Ben-Avraham Z., Kelty T., Yu H.-S. (2014). Origin of marginal basins of the NW Pacific and their plate tectonic reconstructions. Earth-Sci. Rev..

[28] Lu H.F., Sun X.M., Zhang M. (2011). Mineralogy and Geochemistry of NGH Deposits in the South China Sea.

[29] Wang P.C., Li S.Z., Suo Y.H., Guo L.L., Wang G.Z., Hui G.G., Santosh M., Somerville I.D., Cao X.Z., Li Y. (2020). Plate tectonic control on the formation and tectonic migration of Cenozoic basins in northern margin of the South China Sea. Geosci. Front..

[30] Yu H.S. (1990). The Pearl River mouth basin: A rift basin and its geodynamic relationship with the southeastern Eurasian margin. Tectonophysics.

[31] Pang X., Chen C.M., Peng D.J., Zhu M., Shu Y., He M., Shen J., Liu B.J. (2007). Sequence Stratigraphy of Deep-water Fan System of Pearl River, South China Sea. Earth Sci. Front..

[32] Wu N.Y., Yang S.X., Wang H.B. (2009). Gas-bearing fluid in flux sub-system for gas hydrate geological system in Shenhu area, northern South China Sea. Chin. J. Geophys..

[33] McDonnell S.L., Maxb M.D., Cherkisa N.Z., Czarneckia M.F. (2000). Tectono-sedimentary controls on the likelihood of gas hydrate occurrence near Taiwan. Mar. Pet. Geol..

[34] Shyu C.-T., Hsu S.-K., Liu C.-S. (1998). Heat flows off southwest taiwan: Measurements over mud diapirs and estimated from bottom simulating reflectors. Terr. Atmos. Ocean..

[35] Wu S., Zhang G., Huang Y., Liang J., Wong H.K. (2005). Gas hydrate occurrence on the continental slope of the northern South China Sea. Mar. Pet. Geol..

[36] He J.X., Yan W., Zhu Y.H., Zhang W., Gong F.X., Liu S.L., Zhang J.R., Gong X.F. (2013). Bio-genetic and sub-biogenetic gas resource potential and genetic types of natural gas hydrates in the northern marginal basins of South China Sea. Nat. Gas Ind..

[37] Li Y.J., Jiang Z.L., Liang S., Zhu J.Z., Huang Y.P., Luan T.S. (2015). Hydrocarbon Generation in the Lacustrine Mudstones of the Wenchang Formation in the Baiyun Sag of the Pearl River Mouth Basin, Northern South China Sea. Energy Fuels.

[38] Shi H.S., He M., Zhang L.L., Yu Q.H., Pang X., Zhong Z.H., Liu L.H. (2014). Hydrocarbon geology, accumulation pattern and the next exploration strategy in the east ern Pearl River Mouth basin. China Offshore Oil Gas.

[39] Dai J., Ni Y., Huang S., Peng W., Han W., Gong D., Wei W. (2017). Genetic types of gas hydrates in China. Pet. Explor. Dev..

[40] He J., Chen S., Liu H., Liu S. (2009). Natural gas genetic types and source rocks in the northern slope of Baiyun Sag to Panyu Low Uplift in Pearl River Mouth Basin. Acta Pet. Sin..

[41] Zhu J.Z., Shi H.S., He M., Pang X., Yang S.K., Li Z.W. (2008). Origins and Geochemical Characteristics of Gases in LW3-1-1 Wellin the Deep Sea Region of Baiyun Sag, Pearl River Mouth Basin. Nat. Gas Ind..

[42] Zhang S.L., Chen D.F., Huang J.Q. (2007). Conditions of accumulation of gas hydrate in Baiyun Sag. Nat. Gas Ind..

[43] Wu N.Y., Zhang H.Q., Yang S.X., Liang J.Q., Wang H.B. (2007). Preliminary discussion on natural Gas Hydrate (NGH) reservoir system of Shenhu Area, north slope of South China Sea. Nat. Gas Ind..

[44] Yang S.X., Zhang H.Q., Wu N.Y., Su X., Schultheiss P., Holland M., Zhang G.X., Liang J.Q., Lu J.A., Rose K. High concentration hydrate in disseminated forms obtained in Shenhu area, north slope of South China Sea. Proceedings of the 6th International Conference on Gas Hydrates (ICGH 2008).

[45] Fu S.Y., Lu J.A. (2010). The characteristics and origin of gas hydrate in Shenhu area, South China Sea. Mar. Geol. Lett..

[46] Zhang H.Q., Yang S.X., Wu N.Y., Xu X.M., Holland M., Schultheiss P., Rose K., Butler H., Humphrey G., Team G.-S. (2007). Successful and surprising results for China’s first gas hydrate drilling expedition. Fire in the Ice. Methane Hydrate Newsl. Natl. Energy Technol. Lab. US Dep. Energy.

[47] Zhang W., Liang J.Q., Lu J.A., Wei J.G., Su P.B., Fang Y.X., Guo Y.Q., Yang S.X., Zhang G.X. (2017). Accumulation features and mechanisms of high saturation natural gas hydrate in Shenhu Area, northern South China Sea. Petrol. Explor. Dev..

[48] Ten Haven H.L., De Leeuw J.W., Rullkotter J., Damste J.S.S. (1987). Restricted utility of the pristane/phytane ratio as a palaeoenvironmental indicator. Nature.

[49] Powell T.G., McKirdy D.M. (1973). Relationship between Ratio of Pristane to Phytane, Crude Oil Composition and Geological Environment in Australia. Nat. Phys. Sci..

[50] Volkman J.K. (1986). A review of sterol markers for marine and terrigenous organic matter. Org. Geochem..

[51] Peters K.E., Moldowan J.M. (1993). The Biomarker Guide: Interpreting Molecular Fossils in Petroleum and Ancient Sediments.

[52] Volkman J.K., Holdsworth D.G., Neill G.P., Bavor H.J. (1992). Identification of natural, anthropogenic and petroleum hydrocarbons in aquatic sediments. Sci. Total Environ..

[53] Baas M., Pancost R., Geel B.V., Damste J.S.S. (2000). A comparative study of lipids in Sphagnum species. Org. Geochem..

[54] Tolosa I., Fiorini S., Gasser B., Martín J., Miquel J.C. (2013). Carbon sources in suspended particles and surface sediments from the Beaufort Sea revealed by molecular lipid biomarkers and compound-specific isotope analysis. Biogeosciences.

[55] Qiao S., Su M., Kuang Z., Yang R., Liang J., Wu N. (2015). Canyon-related undulation structures in the Shenhu area, northern South China Sea. Mar. Geophys. Res..

[56] Jia Y., Tian Z., Shi X., Liu J.P., Chen J., Liu X., Ye R., Ren Z., Tian J. (2019). Deep-sea Sediment Resuspension by Internal Solitary Waves in the Northern South China Sea. Sci. Rep..

[57] Li X.S., Zhou Q.J., Su T.Y., Liu L.J., Gao S., Zhou S.W. (2016). Slope-confined submarine canyons in the Baiyun deep-water area, northern South China Sea: Variation in their modern morphology. Mar. Geophys. Res..

[58] Lü C., Yao Y., Gong Y., Wu S., Li X. (2012). Deepwater canyons reworked by bottom currents: Sedimentary evolution and genetic model. J. Earth Sci..

[59] Palamenghi L., Keil H., Spiess V. (2014). Sequence stratigraphic framework of a mixed turbidite-contourite depositional system along the NW slope of the South China Sea. Geo-Mar. Lett..

[60] Wang X., Wang Y., Tan M., Cai F. (2020). Deep-water deposition in response to sea-level fluctuations in the past 30 kyr on the northern margin of the South China Sea. Deep. Sea Res. Part I Oceanogr. Res. Pap..

[61] Nishimura M., Baker E.W. (1986). Possible origin of n-alkanes with a remarkable even-to-odd predominance in recent marine sediments. Geochim. Cosmochim. Acta.

[62] Grimalt J., Albaiges J. (1987). Sources and occurrence of C12-C22 n-alkane distributions with even carbon-number preference in sedimentary environments. Geochim. Cosmochim. Acta.

[63] Harji R.R., Yvenat A., Bhosle N.B. (2008). Sources of hydrocarbons in sediments of the Mandovi estuary and the Marmugoa harbour, west coast of India. Environ. Int..

[64] Zaghden H., Kallel M., Elleuch B., Oudot J., Saliot A. (2007). Sources and distribution of aliphatic and polyaromatic hydrocarbons in sediments of Sfax, Tunisia, Mediterranean Sea. Mar. Chem..

[65] Zhu X., Mao S., Sun Y., Jia G., Wu N., Wu D., Guan H., Yan W. (2018). Organic molecular evidence of seafloor hydrocarbon seepage in sedimentary intervals down a core in the northern South China Sea. J. Asian Earth Sci..

[66] Welte D.H., Ebhardt G. (1968). Distribution of long chain n-paraffins and n-fatty acids in sediments from the Persian Gulf. Geochem. Notes.

[67] Lichtfouse E., Bardoux G., Mariotti A., Balesdent J., Ballentine D.C., Makao S.A. (1997). Molecular, 13C, and 14C evidence for the allochthonous and ancient origin of C16-C18 n-alkanes in modern soils. Geochim. Cosmochim. Acta.

[68] Wiesenberg G.L.B., Lehndorff E., Schwark L. (2009). Thermal degradation of rye and maize straw: Lipid pattern changes as a function of temperature. Org. Geochem..

[69] Nguyen Tu T.T., Derenne S.L.C., Mariotti A., Bocherens H., Pons D. (2000). Effects of fungal infection on lipid extract composition of higher plant remains: Comparison of shoots of a Cenomanian conifer, uninfected and infected by extinct fungi. Org. Geochem..

[70] Han J., McCarthy E.D., Van Hoeven W., Calvin M., Bradley W.H. (1968). Organic Geochemical Studies, II. A Preliminary Report on the Distribution of Aliphatic Hydrocarbons in Algae, in Bacteria, and In a Recent Lake Sediment. Proc. Natl. Acad. Sci. USA.

[71] Eglinton G., Hamilton R.J. (1967). Leaf Epicuticular Waxes. Science.

[72] Jones J.G. (1969). Studies on Lipids of Soil Micro-organisms with Particular Reference to Hydrocarbons. J. Gen. Microbiol..

[73] Grimalt J., Albaiges J., Alexander G.H.I. (1986). Predominance of Even Carbon-Numbered n-Alkanes in Coal Seam Samples of Nograd Basin (Hungary). Naturwissenschaften.

[74] Grimalt J., Albaiges J., A1-Saad H.T., Douabul A.A.Z. (1985). n-Alkane Distributions in Surface Sediments from the Arabian Gulf. Naturwissenschaften.

[75] Grimalt J., Torras E., Albaiges J. (1987). Bacterial reworking of sedimentary fipids during sample storage. Org. Geochem..

[76] Aceves M., Grimaltrk J.O. (1992). Gas chromatographic screening of organic compounds in urban aerosols: Selectivity effects in semi-polar columns—ScienceDirect. J. Chromatogr..

[77] Villanueva J., Grimalt J., Cortijo E., Vidal L., Labeyrie L. (1997). A biomarker approach to the organic matter deposited in the North Atlantic during the last climatic cycle. Geochim. Cosmochim. Acta.

[78] Bray E.E., Evans E.D. (1961). Distribution of n-paraffins as a clue to recognition of source beds. Geochim. Cosmochim. Acta.

[79] Huang W.Y., Meinschein W.G. (1979). Sterols as ecological indicators. Geochim. Cosmochim. Acta.

[80] Shi J.Y., Mackenzie A.S., Alexander R., Eglinton G., Gowar A.P., Wolff G.A., Maxwell J.R. (1982). A biological marker investigation of petroleums and shales from the Shengli oilfield, The People’s Republic of China. Chem. Geol..

[81] Seifert W.K., Moldowan J.M. (1980). The effect of thermal stress on source-rock quality as measured by hopane stereochemistry. Phys. Chem. Earth.

[82] Mackenzie A.S., Brooks S.J., Welte D. (1984). Applications of biological markers in petroleum geochemistry. Advances in Petrological Geochemistry.

[83] Espitalie J., Madec M., Tissot B. Source rock characterization method for petroleum exploration. Proceedings of the Offshore Technology Conference, OTC.

[84] Tissot B.P., Welte D.H. (1984). Petroleum Formation and Occurrence.

[85] Peters K.E. (1986). Guidelines for Evaluating Petroleum Source Rock Using Programmed Pyrolysis. AAPG Bull..

[86] Stein R. (1991). Accumulation of Organic Carbon in Marine Sediments.

[87] Calvert S.E. (2004). Beware intercepts: Interpreting compositional ratios in multi-component sediments and sedimentary rocks. Org. Geochem..

[88] Katz B.J. (1983). Limitations of ‘Rock-Eval’ pyrolysis for typing organic matter. Org. Geochem..

[89] Espitalie J., Madec M., Tissot B. (1980). Role of Mineral Matrix in Kerogen Pyrolysis: Generation and Migration. Am. Assoc. Pet. Geol. Bull..

[90] Dang Y., Zhao W., Su A., Zhang S., Li M., Guan Z., Ma D., Chen X., Shuai Y., Wang H. (2008). Biogenic gas systems in eastern Qaidam Basin. Mar. Pet. Geol..

[91] Kotelnikova S. (2002). Microbial production and oxidation of methane in deep subsurface. Earth-Sci. Rev..

[92] Shuai Y., Zhang S., Grasby S.E., Chen Z., Ma D., Wang L., Li Z., Wei C. (2013). Controls on biogenic gas formation in the Qaidam Basin, northwestern China. Chem. Geol..

[93] Rice D.D., Claypool G.E. (1981). Generation, Accumulation, and Resource Potential of Biogenic Gas. AAPG Bull..

[94] Rashid M.A., Vilks G. (1977). Environmental controls of methane production in Holocene basins in eastern Canada. Org. Geochem..

[95] Waseda A. (1998). Organic carbon content, bacterial methanogenesis, accumulation and processes of gas hydrates in marine sediments. Geochem. J..

[96] Wallmann K., Pinero E., Burwicz E., Haeckel M., Hensen C., Dale A., Ruepke L. (2012). The Global Inventory of Methane Hydrate in Marine Sediments: A Theoretical Approach. Energies.

[97] Peters K.E., Cassa M.R., Magoon L.B., Dow W.G. (1994). Applied Source-Rock Geochemistry. The Petroleum System. From Source to Trap.

[98] Blumer M., Sass J. (1972). Oil Pollution: Persistence and Degradation of Spilled Fuel Oil. Science.

[99] Lytle J.S., Lytle T.F., Gearing J.N., Gearing P.J. (1979). Hydrocarbons in benthic algae from the Eastern Gulf of Mexico. Mar. Biol..

[100] Mesle M., Dromart G., Oger P. (2013). Microbial methanogenesis in subsurface oil and coal. Res. Microbiol..

[101] Bouloubassi I., Fillaux J., Saliot A. (2001). Hydrocarbons in Surface Sediments from the Changjiang (Yangtze River) Estuary, East China Sea. Mar. Pollut. Bull..

[102] Grantham P.J. (1986). Steranes isomerization and moretane/hopane ratios in crude oils derived from tertiary source rocks. Org. Geochem..

[103] Ramon J.C., Dzou I.L. (1999). Petroleum geochemistry of Middle Magdalena Valley, Colombia. Organic Geochem..

[104] Huang D.F., Li J.C., Zhang D.J., Huang X.M., Zhou Z.H. (1991). Maturation sequence of tertiary crude oils in the qaidam basin and its significance in petroleum resource assessment. J. Asian Earth Sci..

[105] Lewan M.D., Bjorøy M., Dolcater D.L. (1986). Effects of thermal maturation on steroid hydrocarbons as determined by hydrous pyrolysis of phosphoria retort shale. Geochim. Cosmochim. Acta.

[106] Seifert W.K., Moldowan J.M. (1986). Use of biological markers in petroleum exploration; Utilisation de marqueurs biologiques dans la prospection de pétrole. Methods Geochem. Geophys..

[107] Kvenvolden K.A., Hostettler F.D., Carlson R.R., Rapp J.B., Threlkeld C.N., Warden A. (1995). Ubiquitous tar balls with a California-source signature on the shorelines of Prince William Sound. Alaska. Environ. Sci. Technol..

[108] Li M., Yao H., Fowler M.G., Stasiuk L.D., Horsfield B., Radke M. (1998). Geochemical constraints on models for secondary petroleum migration along the upper devonian rimbey-meadowbrook reef trend in central alberta, canada. Org. Geochem..

[109] Hanson A.D., Zhang S.C., Moldowan J.M., Liang D.G., Zhang B.M. (2000). Molecular Organic Geochemistry of the Tarim Basin, Northwest China. AAPG Bull..

[110] Waples D.W., Machihara T. (1990). Application of sterane and triterpane biomarkers in petroleum exploration. Bull. Can. Pet. Geol..

[111] Rullkotter J., Marzi R. (1988). Natural and artificial maturation of biological markers in a Toarcian shale from northern Germany. Org. Geochem..

[112] Waples D.W. (1985). Geochemistry in Petroleum Exploration.

[113] Pan C.C., Peng D.H., Zhang M., Yu L.P., Sheng G.Y., Fu J.M. (2008). Distribution and isomerization of C31–C35 homohopanes and C29 steranes in oligocene saline lacustrine sediments from Qaidam Basin, Northwest China. Org. Geochem..

[114] Peters K.E., Walters C.C., Moldowan J.M. (2005). The Biomarker Guide: Biomarkers and Isotopes in Petroleum Exploration and Earth History.

[115] Shanmugam G. (1985). Significance of coniferous rain forests and related organic matter in generating commercial quantities of oil, Gippsland Basin, Australia. AAPG (Am. Assoc. Pet. Geol.) Bull..

[116] Jiang W., Li Y., Yang C., Xiong Y. (2021). Organic geochemistry of source rocks in the Baiyun Sag of the Pearl River Mouth Basin, South China Sea. Mar. Pet. Geol..

[117] Niu Z., Liu G., Ge J., Zhang X., Cao Z., Lei Y., An Y., Zhang M. (2019). Geochemical characteristics and depositional environment of Paleogene lacustrine source rocks in the Lufeng Sag, Pearl River Mouth basin, South China Sea. J. Asian Earth Sci..

[118] Peng J., Pang X., Peng H., Ma X., Shi H., Zhao Z., Xiao S., Zhu J. (2017). Geochemistry, origin, and accumulation of petroleum in the Eocene Wenchang Formation reservoirs in Pearl River Mouth Basin, South China Sea: A case study of HZ25-7 oil field. Mar. Pet. Geol..

[119] Cheng P., Tian H., Huang B.J., Wilkins R.W.T., Xiao X.M. (2013). Tracing early-charged oils and exploration directions for the Wenchang A sag, western Pearl River Mouth Basin, offshore South China Sea. Org. Geochem..

[120] Ping H., Chen H., Zhu J., George S.C., Mi L., Pang X., Zhai P. (2018). Origin, source, mixing, and thermal maturity of natural gases in the Panyu lower uplift and the Baiyun depression, Pearl River Mouth Basin, northern South China Sea. AAPG Bull..

[121] Ping H., Chen H., Zhai P., Zhu J., George S.C. (2019). Petroleum charge history in the Baiyun depression and Panyu lower uplift in the Pearl River Mouth Basin, northern South China Sea: Constraints from integration of organic geochemical and fluid inclusion data. AAPG Bull..

[122] Zhang L.L., Shu Y., Cai G.F., Long Z.L., Liu D.Q., Wang F. (2019). Eocene-Oligocene sedimentary environment evolution and its impact on hydrocarbon source conditions in eastern Pearl River Mouth Basin. Acta Pet. Sin..

[123] Pang X., Chen C.M., Zhu M., He M., Shen J., Liu B.J. (2006). A discussion about hydrocarbon accumulation conditions in Baiyun Deep-water Area, the northern continental slope, South China Sea. China Offshore Oil Gas.

[124] Su P.B., Lei H.Y., Liang J.Q., Sha Z.B., Fu S.Y., Gong Y.H. (2010). Characteristics of gas source in the waters of Shenhu and their significances to gas hydrate accumulation. Nat. Gas Ind..

[125] Schlegel M.E., McIntosh J.C., Petsch S.T., Orem W.H., Jones E.J.P., Martini A.M. (2013). Extent and limits of biodegradation by in situ methanogenic consortia in shale and formation fluids. Appl. Geochem..

[126] Knittel K., Boetius A. (2009). Anaerobic oxidation of methane: Progress with an unknown process. Annu. Rev. Microbiol..

[127] Milkov A.V. (2011). Worldwide distribution and significance of secondary microbial methane formed during petroleum biodegradation in conventional reservoirs. Org. Geochem..

[128] Pape T., Bahr A., Rethemeyer J., Kessler J.D., Sahling H., Hinrichs K.-U., Klapp S.A., Reeburgh W.S., Bohrmann G. (2010). Molecular and isotopic partitioning of low-molecular-weight hydrocarbons during migration and gas hydrate precipitation in deposits of a high-flux seepage site. Chem. Geol..

[129] Gong J., Sun X., Xu L., Lu H. (2017). Contribution of thermogenic organic matter to the formation of biogenic gas hydrate: Evidence from geochemical and microbial characteristics of hydrate-containing sediments in the Taixinan Basin, South China Sea. Mar. Pet. Geol..

[130] Milkov A.V., Etiope G. (2018). Revised genetic diagrams for natural gases based on a global dataset of >20,000 samples. Org. Geochem..

