# Supplementary material for: Geochemical Characteristics and Significance of Organic Matter in Hydrate-Bearing Sediments from Shenhu Area, South China Sea
Source: Molecules. 2022 Apr 14;27(8):2533. doi: 10.3390/molecules27082533 (PMC9025314; doi:10.3390/molecules27082533)
Supplement: Supplementary file 1 [file molecules-27-02533-s001.zip › molecules-1636117-supplementary.pdf]

Table S1. Site information and lithological description of the samples in this study.

Site	Water Depth	Drilling depth	Geothermal gradient	Hydrate-bearing layer	Max hydrate saturation	Gas source	Sample No.	Depth	Lithology
	(mbsf)	(mbsf)	(°C/km)	(mbsf)	%			(mbsf)	
W01B	1285	234	64.90	147-172	61.90	Thermogenic and Mixed	1B-2H	54.90	Fine Silt
							1B-4H	111.90	Very Fine Silt
							1B-5H	119.52	Very Fine Silt
							1B-6H	127.90	Fine Silt
							1B-7X	134.15	Medium Silt
							1B-10X	145.48	Foram-rich Medium Silt
							1B-16X	165.77	Medium Silt
							1B-17X	166.88	Medium Silt
							1B-20X	178.26	Medium Silt
							1B-23X	192.77	Fine Silt
W02B	1274	240	61.20	140-175	49.90	Thermogenic and Mixed	2B-1H	44.90	Very Fine Silt
							2B-3H	83.90	Very Fine Silt
							2B-4H	99.90	Very Fine Silt
							2B-6H	106.86	Foram-rich Fine Silt
							2B-7H	116.74	Foram-rich Fine Silt
							2B-9H	128.69	Foram-rich Medium Silt
							2B-11X	137.41	Fine Silt
							2B-16X	153.85	Medium Silt
							2B-19X	166.01	Medium Silt
							2B-21X	173.64	Fine Silt
							2B-22X	176.81	Medium Silt
							2B-25X	189.90	Very Fine Silt
W03B	1310	222	54.60	140-210	53.00	Biogenic and Mixed	3B-1H	47.90	Medium Silt
							3B-3X	94.43	Medium Silt
							3B-6X	131.90	Fine Silt
							3B-11X	149.43	Fine Silt
							3B-12X	154.15	Fine Silt
							3B-17X	170.85	Fine Silt
							3B-18X	176.36	Medium Silt
							3B-22X	190.65	Fine Silt
							3B-24X	199.47	Fine Silt
							3B-25X	204.28	Fine Silt
							3B-26X	210.08	Fine Silt
							3B-27X	213.90	Fine Silt
							3B-28X	218.00	Fine Silt

Table S2. Biomarker composition and Rock-Eval pyrolysis parameters of sediments in this study.

Sample NO.	Depth/mbsf	Chloroform "A" (%)	Aliphatic (%)	Aromatic (%)	TOC (%)	Σn C21-/Σn C21+	OEP1	OEP2	Squalane	Pre-peak/Post-peak	Pr/Ph	Pr/nC_{17}	Ph/nC_{18}	TAR	Sterane ∑C27/∑C29	Sterane C29ββ/ββ+αα	Sterane C29αα20S/20(S+R)	Ts/Tm	Hopane C31αβ22S/22(S+R)	S1(mg/g)	S2(mg/g)	Tmax(℃)	S3CO(mg/g)	PC(%)	RC(%)	TOC(%)	HI	OI
**1B-2H**	**54.90**	**0.03**	**13.45**	**5.65**	**1.17**	**0.19**	**0.31**	**8.33**		**0.17**	**0.80**	**0.62**	**0.22**	**16.83**						**0.13**	**1.03**	**404.00**	**0.68**	**0.18**	**1.16**	**1.34**	**77.00**	101.00
1B-4H	111.90	0.00	17.69	7.07	0.27	0.50	0.19	4.34	5.85	1.06	0.60	0.22	0.11	4.16	0.78	0.31	0.29	0.63	0.39	0.23	0.13	331.00	0.25	0.05	0.27	0.32	41.00	91.00
1B-5H	119.52	0.01	24.25	7.00	0.26	0.95	0.22	4.22	2.73	1.98	0.71	0.28	0.19	3.29	0.61	0.30	0.43	0.51	0.53	0.11	0.09	337.00	0.26	0.04	0.24	0.28	32.00	125.00
1B-6H	127.90	0.01	24.38	7.06	0.36	0.60	0.26	3.62	5.16	1.09	0.94	0.33	0.12	4.86	0.70	0.24	0.19	0.44	0.41	0.13	0.10	344.00	0.20	0.04	0.25	0.29	34.00	86.00
1B-7X	134.15	0.01	26.57	6.79	0.30	1.06	0.29	3.89	5.16	1.97	0.77	0.63	0.34	2.36	0.44	0.36	0.25	0.49	0.43	0.20	0.10	338.00	0.08	0.04	0.19	0.23	43.00	91.00
1B-10X	145.48	0.01	40.11	6.87	0.20	2.69	0.51	2.13	1.58	5.76	1.11	0.48	0.32	0.48	0.58	0.42	0.39	0.77	0.55	0.12	0.09	333.00	0.28	0.04	0.27	0.31	29.00	116.00
1B-16X	165.77	0.02	0.19	0.85	0.44	0.64	0.45	5.48	10.06	1.38	0.76	0.50	0.24	1.33	0.55	0.32	0.21	0.81	0.33	0.07	0.04	346.00	0.14	0.02	0.19	0.21	19.00	90.00
1B-17X	166.88	0.01	36.56	2.92	0.36	4.35	0.47	3.20	1.38	9.27	1.04	0.69	0.16	0.30	0.64	0.29	0.27	0.44	0.32	0.06	0.01	345.00	0.09	0.01	0.14	0.15	7.00	40.00
1B-20X	178.26	0.00	23.60	6.56	0.31	0.77	0.25	3.60	3.57	1.33	0.62	0.36	0.19	4.03	0.65	0.30	0.16	0.25	0.24	0.10	0.05	343.00	0.11	0.02	0.19	0.21	24.00	76.00
1B-23X	192.77	0.01	29.94	5.15	0.21	1.22	0.54	2.57	3.20	2.00	0.82	0.52	0.39	1.25	0.83	0.40	0.26	0.56	0.23	0.04	0.02	362.00	0.07	0.01	0.19	0.20	10.00	65.00
2B-1H	44.90	0.01	12.99	3.14	0.77	0.42	0.36	4.26	7.84	0.50	0.79	0.79	0.29	5.96	0.86	0.36	0.28	0.38	0.35	0.08	0.46	394.00	0.66	0.11	0.73	0.84	55.00	121.00
2B-3H	83.90	0.01	23.28	4.64	0.56	0.80	0.19	8.40	2.56	1.29	0.70	0.44	0.14	5.14	0.71	0.31	0.32	0.54	0.38	0.07	0.20	391.00	0.44	0.06	0.54	0.60	33.00	108.00
2B-4H	99.90	0.01	24.53	7.25	0.40	0.93	0.11	5.50	3.70	1.95	0.64	0.29	0.08	6.46	0.79	0.33	0.31	0.57	0.30	0.12	0.18	399.00	0.44	0.06	0.40	0.46	39.00	139.00
2B-6H	106.86	0.01	29.68	8.71	0.49	0.51	0.38	4.15	4.93	1.02	0.97	0.20	0.21	4.68	0.35	0.32	0.33	0.46	0.39	0.06	0.10	388.00	0.28	0.04	0.34	0.38	26.00	100.00
2B-7H	116.74	0.00	28.33	6.87	0.37	0.73	0.16	5.19	4.03	1.17	0.75	0.57	0.11	6.75	0.39	0.43	0.39	0.36	0.40	0.06	0.08	398.00	0.25	0.03	0.26	0.29	28.00	121.00
2B-9H	128.69	0.01	23.51	9.65	0.36	1.29	0.20	3.42	5.03	2.23	0.61	0.51	0.20	2.21	0.17	0.28	0.15	0.36	0.29	0.06	0.12	388.00	0.22	0.03	0.24	0.27	44.00	111.00
2B-11X	137.41	0.00	33.25	5.06	0.30	0.86	0.25	4.14	4.68	1.44	0.72	0.25	0.17	3.66	0.42	0.28	0.18	0.25	0.33	0.07	0.08	394.00	0.18	0.03	0.18	0.21	38.00	110.00
2B-16X	153.85	0.00	0.89	14.24	0.23	0.07	0.10	2.42	2.49	1.58	0.75	0.72	0.09	1.48	0.40	0.32	0.19	0.72	0.26	0.05	0.05	388.00	0.16	0.02	0.19	0.21	24.00	110.00
2B-19X	166.01	0.01	29.73	6.86	0.18	1.44	0.20	3.87	3.50	2.49	0.67	0.32	0.39	2.47	0.66	0.41	0.36	0.69	0.37	0.07	0.05	377.00	0.12	0.02	0.13	0.15	33.00	107.00
2B-21X	173.64	0.01	27.16	7.79	0.22	0.50	0.12	3.83	3.42	1.01	0.87	0.53	0.09	11.25	0.75	0.30	0.21	0.29	0.26	0.04	0.05	375.00	0.16	0.02	0.19	0.21	24.00	105.00
2B-22X	176.81	0.01	10.99	4.79	0.18	1.39	0.26	5.12	3.24	2.41	0.76	0.35	0.21	1.73	0.59	0.29	0.24	0.19	0.21	0.03	0.03	377.00	0.15	0.02	0.17	0.19	16.00	105.00
2B-25X	189.90	0.01	17.45	6.28	0.27	1.19	0.32	3.17	5.92	1.86	0.95	0.49	0.31	1.80	0.98	0.23	0.28	0.52	0.25	0.05	0.05	366.00	0.18	0.02	0.22	0.24	21.00	104.00
3B-1H	47.90	0.00	27.67	4.33	0.04	1.92	0.35	3.40	3.71	3.35	0.69	0.36	0.30	0.98	1.37	0.49	0.24	0.42	0.21	0.04	0.05	393.00	0.08	0.02	0.19	0.21	24.00	86.00
3B-3X	94.43	0.00	14.76	5.09	0.07	1.58	0.24	3.12	6.70	2.51	0.76	0.51	0.26	1.91	0.89	0.38	0.08	0.55	0.17	0.03	0.03	395.00	0.06	0.01	0.18	0.19	16.00	74.00
3B-6X	131.90	0.02	19.66	5.26	0.07	1.21	0.24	4.06	6.66	1.75	0.72	0.55	0.26	2.69	1.00	0.19	0.03	0.16	0.16	0.05	0.05	380.00	0.13	0.02	0.21	0.23	22.00	78.00
3B-11X	149.43	0.01	23.83	5.62	0.09	1.26	0.22	3.53	6.42	2.08	0.80	0.66	0.28	2.53	1.47	0.17	0.03	0.11	0.16	0.05	0.07	403.00	0.09	0.02	0.24	0.26	27.00	81.00
3B-12X	154.15	0.01	0.97	7.99	0.08	0.29	0.39	1.85	10.58	0.52	0.18	0.39	0.50	3.92	0.82	0.23	0.14	0.19	0.10	0.06	0.08	374.00	0.11	0.03	0.27	0.30	27.00	80.00
3B-17X	170.85	0.01	32.91	4.75	0.12	1.07	0.30	3.53	5.13	1.43	0.74	0.54	0.34	2.37	1.04	0.12	0.04	0.31	0.21	0.03	0.11	400.00	0.11	0.03	0.29	0.32	34.00	78.00
3B-18X	176.36	0.00	20.85	4.86	0.08	0.97	0.37	3.46	6.00	1.19	0.63	0.53	0.45	1.99	0.93	0.10	0.01	0.25	0.24	0.03	0.09	400.00	0.09	0.02	0.24	0.26	35.00	88.00

3B-22X	190.65	0.01	17.70	5.83	0.15	0.98	0.25	3.87	10.44	1.35	0.55	0.53	0.40	3.12	1.00	0.10	0.01	0.22	0.19	0.04	0.13	402.00	0.12	0.03	0.26	0.29	45.00	93.00
3B-24X	199.47	0.00	14.48	4.62	0.15	0.66	0.47	3.43	13.19	0.74	0.54	0.69	0.87	2.74	1.01	0.07	0.01	0.13	0.19	0.05	0.11	403.00	0.10	0.03	0.26	0.29	38.00	79.00
3B-25X	204.28	0.00	31.62	3.60	0.15	0.48	0.56	3.30	8.01	0.55	0.43	0.61	1.11	3.49	1.07	0.05	0.00	0.12	0.21	0.04	0.08	400.00	0.15	0.02	0.25	0.27	30.00	78.00
3B-26X	210.08	0.00	43.67	3.32	0.22	0.51	0.48	3.26	6.90	0.72	0.31	0.48	0.93	3.49	1.06	0.13	0.01	0.20	0.21	0.01	0.08	405.00	0.10	0.02	0.28	0.30	27.00	77.00
3B-27X	213.90	0.00	39.43	4.96	0.26	1.26	0.40	5.16	6.05	1.56	0.63	0.35	0.51	1.86	0.99	0.15	0.05	0.38	0.16	0.04	0.08	391.00	0.09	0.02	0.24	0.26	31.00	81.00
3B-28X	218.00	0.00	33.61	4.78	0.20	0.60	0.51	3.00	4.28	0.71	0.78	0.54	0.56	3.12						0.06	0.09	380.00	0.10	0.03	0.26	0.29	31.00	76.00
